# *In vitro* and clinical data demonstrate negligible risk of drug–drug interactions with opelconazole, a novel inhaled antifungal agent

**DOI:** 10.1093/jac/dkaf384

**Published:** 2025-10-17

**Authors:** Lindsey M R Cass, John Ayrton, Roger J Brüggemann, Jerome Moore, Ubaldo Martin, Laura Grey, Michele Hyman, Lance Berman

**Affiliations:** Pulmocide Ltd., 44 Southampton Buildings, London WC2A 1AP, UK; Pulmocide Ltd., 44 Southampton Buildings, London WC2A 1AP, UK; Antifungal Pharmacology Research Group, Radboud University Medical Centre, Nijmegen, The Netherlands; Pacific BioDevelopment, LLC, Davis, CA, USA; Pulmovant, Inc., Waltham, MA, USA; S-cubed Biometrics, Oxford, UK; BCH Research Solutions, LLC, Plymouth Meeting, PA, USA; Pulmocide Ltd., 44 Southampton Buildings, London WC2A 1AP, UK

## Abstract

**Background:**

Invasive fungal diseases (IFDs) cause high morbidity and mortality among immune-compromised patients. Triazoles, which are recommended to treat IFDs, inhibit and/or are substrates for the phase 1 cytochrome P450 (CYP) pathway that metabolizes many drugs and frequently result in drug–drug interactions (DDIs). Nebulized opelconazole is an investigational inhaled antifungal in development for the treatment of pulmonary aspergillosis.

**Methods:**

*In vitro* studies first assessed effects of opelconazole on CYP isoforms and human transporter protein interactions. Subsequently, a Phase 1 study in healthy volunteers assessed whether repeat daily dosing of inhaled opelconazole inhibited or induced CYP1A2 and CYP3A4 using the substrates caffeine and midazolam, respectively.

**Results:**

*In vitro* analyses confirmed that opelconazole interactions with human CYPs were limited to competitive inhibition of CYP3A4 and weak induction potential for CYP3A4 and CYP1A2. These interactions occurred at concentrations higher than those expected at steady state following clinical dosing. Inhibition of a limited range of transporter proteins occurred at micromolar concentrations of opelconazole, but it was not found to be a substrate for carrier transporters. In the Phase 1 healthy volunteer study, inhaled opelconazole at steady state demonstrated no inhibition/induction of CYP3A4 or CYP1A2 and was generally well tolerated. The Phase 1 study results confirmed that the potential for interactions of opelconazole with CYP3A4 and CYP1A2 substrates during concomitant use was negligible.

**Conclusions:**

Systemic steady-state concentrations achieved after clinical dosing of inhaled opelconazole are unlikely to be associated with CYP-mediated DDIs or transporter-mediated DDIs.

## Introduction

Invasive aspergillosis (IA) occurs in 4% of patients undergoing remission induction chemotherapy for haematologic malignancies, 10% of allogeneic haematologic stem cell transplant recipients and 3%–19% of lung transplant recipients, despite antifungal prophylaxis.^1–3^ It also occurs in patients treated with prolonged high-dose corticosteroids, other immunosuppressants (notably in lung transplant recipients), or targeted cancer therapies, those with chronic pulmonary disease, lung cancer, hepatic cirrhosis or in critically ill patients requiring hospitalization following influenza or COVID-19.^2–8^

Systemic antifungals are guideline-recommended therapies for the treatment and prevention of IA.^9,10^ However, currently available antifungals may have important limitations such as treatment-limiting side effects and drug–drug interactions (DDIs).^8,9^

Triazoles have the highest risk of DDIs amongst antifungals because they both inhibit and/or are substrates for the phase 1 cytochrome P450 (CYP) biotransformation pathway, which is involved in metabolizing and clearing several common drugs.^11,12^ The CYP3A isoenzyme comprises the largest fraction of the CYP content in humans, and the CYP3A4 isoenzyme is inhibited to different degrees by all approved triazoles.^12^ Additionally, triazoles both inhibit and are substrates for P-glycoprotein (P-gp) and organic-anion transporter peptides (OATPs) that are involved in drug absorption and distribution.^12–14^

Existing triazoles are administered orally or intravenously and require high systemic exposure to reach tissue concentrations high enough for pathogen clearance.^13,15–18^ These routes of administration, as well as high systemic concentrations, affect the sites of interaction with CYP3A4 enzymes that are present in the small intestine (oral triazoles) and liver (oral and intravenous triazoles).^13,15,16^ Inhibition of CYP3A4 by triazoles and other drugs, therefore, presents a high risk of clinically relevant DDIs.^13^ Triazoles have a narrow therapeutic window, with systemic concentrations that must be carefully monitored to prevent drug concentrations that are too low for efficacy or too high for tolerability or safety.^9,19^ The DDIs associated with triazoles and concomitant medications can increase or decrease plasma concentrations of the triazole, of the concomitant medication, or both drugs, leading to increased risk of toxicity and/or therapeutic failure.^20^

PC945 (opelconazole) Nebuliser Suspension (hereinafter, opelconazole) is a novel, investigational triazole with a similar antifungal mechanism of action as approved triazoles (inhibits ergosterol synthesis), which has been formulated for inhaled administration.^17,21^ Based on the first-in-human pharmacokinetic (PK) study as well as clinical observations in a limited expanded access programme, opelconazole plasma concentrations of 1–10 ng/mL are expected to indicate the achievement of therapeutic lung concentrations, which would exceed the minimal inhibitory concentration required to inhibit 90% of *Aspergillus fumigatus* clinical strains (1 mg/L), with a long duration of action.^17^ This PK profile of high lung concentrations and low systemic exposure should allow treatment with opelconazole to provide effective antifungal activity in the lung with a reduced risk of systemic side effects.^21,22^ The available opelconazole PK data, indicating slow absorption from the lungs, low systemic exposure [*C*_max_ of 0.0016 μmol/L (1 ng/mL)], and high plasma protein binding (PPB; 96%–98% with a free fraction concentration of 0.03 ng/mL), together with the available safety data indicate limited potential for systemic side effects and DDIs.^17^

As the structure of opelconazole includes a triazole moiety, the compound could show inhibitory interactions with some CYP isoforms. Thus, through *in vitro* studies, we first explored the ability of opelconazole to act as a substrate, inhibitor or inducer of CYP isoforms, and its effects on efflux and uptake transporters. Based on analyses of the opelconazole *in vitro* data, we then conducted a Phase 1 clinical study to examine how opelconazole affected CYP3A4 using midazolam as the probe agent and CYP1A2 using caffeine as the probe agent.

## Materials, participants and methods

### *In vitro* DDI studies

#### Opelconazole as a substrate of human CYP isoforms

The testing of opelconazole as a potential substrate of CYP1A2, 2B6, 2C8, 2C9, 2C19, 2D6 and 3A4 was determined *in vitro* using recombinant CYP enzymes. Two concentrations of opelconazole (0.01 and 0.1 µM) were tested with each of the seven CYPs.

#### Opelconazole as an inhibitor of human CYP isoforms

The potential for opelconazole to inhibit CYP isoforms was tested *in vitro* in human liver microsomes. An initial study, using opelconazole concentrations ranging from 0.03 to 5 μM, showed that CYP inhibition was limited to CYP3A4. In a subsequent *in vitro* study, the IC_50_ of opelconazole was determined using a range of concentrations that reflected the observed magnitude of opelconazole clinical exposure (0.001–0.25 µM). Preincubation of the human liver microsomes for periods of 0 min without NADPH and 30 min with NADPH, plus 30 min without NADPH were included to assess for a time-dependent inhibition effect. Two structurally diverse substrates (testosterone and midazolam) were tested to assess substrate-dependent inhibition.

#### Opelconazole as an inducer of human CYP isoforms

The potential for opelconazole to act as an inducer of CYP isoforms CYP1A2, CYP2B6 and CYP3A4 was assessed *in vitro* using an mRNA endpoint across three donors in cryopreserved human hepatocytes. A drug is interpreted as an inducer if: (1) it increases mRNA expression of a CYP enzyme in a concentration-dependent manner and (2) the fold change of CYP mRNA expression relative to vehicle control is ≥2-fold at the drug’s expected hepatic concentrations.^23^

A cytotoxicity assessment using MTT was performed in one of the three donors used for the induction studies prior to dosing. Using MTT, no cytotoxicity was observed for opelconazole up to 3 μM, hence this concentration was chosen as the maximum test concentration for induction assessment. The hepatocyte cells were dosed with opelconazole (0.012, 0.03, 0.12, 0.3, 1.2 and 3 μM) and positive control compounds (omeprazole for CYP1A2 and rifampicin for CYP3A4). Cells were exposed to the solutions for 72 h with fresh solution added every 24 h. Test compound measurement generally demonstrated good agreement between measured and nominal concentrations at both selected test concentrations (0.3 and 3 μM).

#### Opelconazole as a substrate or inhibitor of carrier transporters

Human transporter protein interactions with opelconazole as a potential substrate and/or inhibitor were assessed for the following key carrier transporters: organic anion transporters, OAT1 and OAT3; organic cation transporters, OCT1 and OCT2; OATP1B1 and OATP1B3; multidrug and toxin extrusion, MATE-1 and MATE2-K; key ATP-binding cassette (ABC) transporters including P-gp, breast cancer resistance protein (BCRP) and bile salt export pump (BSEP). Two concentrations of opelconazole, 0.5 and 5 μM, were tested using MDCKII cells, human embryonic kidney 293 cells and BSEP-transfected sf9 membrane vesicles and then analysed by LC-tandem MS to determine transporter inhibition and whether opelconazole was a transporter substrate. The viability of experimental test systems was confirmed with controls and known probe substrates and probe inhibitors.

The analyses of opelconazole on the CYP isoforms CYP1A2 and CYP3A4 constitute the rationale to study the effects of opelconazole on these isoenzymes with appropriate substrates in a clinical setting.

### Phase 1 DDI study

#### Study design

Following the observations in the *in vitro* studies, a Phase 1, single-centre, open-label study in healthy volunteers was conducted to evaluate the effect of multiple doses of inhaled opelconazole on the PK of midazolam (as the probe substrate for CYP3A4) and caffeine (as the probe substrate for CYP1A2). The study was conducted from February 2023 through June 2023 in accordance with the Declaration of Helsinki, Good Clinical Practice, and the International Conference on Harmonisation guidelines (South African National Clinical Trial Registry code: DOH-27-122022-5196). The study documents were approved by an Independent Ethics Committee. Study participants provided written informed consent prior to any study procedures. Twenty-four healthy, male and female volunteers aged 18–55 years who weighed ≥50 kg and had a BMI of 18.5–30 kg/m^2^ participated (Table 1).

**Table 1.** Demographics and baseline characteristics of Phase 1 study healthy volunteers

		Participants (*N* = 24)
Sex, *n* (%)	Male	16 (66.7)
	Female	8 (33.3)
Mean (SD) age at screening, years		31.2 (7.5)
Mean (SD) height at screening, m		1.7 (0.08)
Mean (SD) weight at screening, kg		68.0 (9.4)
Mean (SD) BMI at screening, kg/m^2^		24.8 (2.8)
Race, *n* (%)	Black or African American*	20 (83.3)
	White	1 (4.2)
	Other	3 (12.5)

^*^Race was self-reported as ‘Black’ by participants; however, these participants were included in the standard race category ‘Black or African American’ during data reporting.

SD, standard deviation.

#### Dosing and sampling schedules

**Figure 1.**
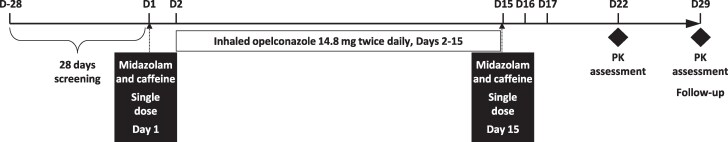
Phase 1 study design. D, day; PK, pharmacokinetic.

Screening occurred within 28 days prior to the first dosing day (Figure 1). Participants were admitted to the study centre on Day −1 and were required to stay there until approximately 48 h after the morning dosing on Day 15. They returned for subsequent PK blood samples at approximately 72 (Day 18), 168 (Day 22) and 336 h (Day 29) after the last morning dosing. On Days 1 and 15, participants received oral midazolam 7.5 mg and oral caffeine 180 mg together after food ingestion.

Standard doses of midazolam and caffeine were used to characterize their PK profiles. The dose selected for opelconazole, 14.8 mg twice daily, is the dose utilized in the clinical development programme for the treatment and prevention of invasive pulmonary aspergillosis (IPA). Following 14 days of twice daily dosing, plasma concentrations were expected to be within 80% of steady-state concentrations and deemed appropriate to assess potential interactions with the probe substrates.

Study participants inhaled opelconazole 14.8 mg twice daily using the PARI LC SPRINT^®^ jet nebuliser for 14 days, starting on Day 2 through Day 15. Morning doses of opelconazole on Day 2 and Day 15 were taken 30 min after food ingestion. Blood samples were taken for PK analyses for midazolam, metabolites of midazolam (1-OH-midazolam and 4-OH-midazolam) and caffeine on Days 1 and 15 at pre-dose (0 h), at 0.5, 1, 1.5, 2, 3, 4, 6, 8, 10, 12, 16, 24, 30, 36 and 48 h post-dose on Days 1 and 15. Blood samples were taken for PK analyses for opelconazole as follows: on Day 2 at pre-morning dose (0 h), at 0.5, 1, 2, 4, 6, 8, 12 and 24 h post-morning dose; on Day 15: at pre-morning-dose (0 h), at 0.5, 1, 2, 4, 6, 8, 10, 12, 24 (Day 16), 30 (Day 16), 36 (Day 16), 48 (Day 17), 72 (Day 18), 168 (Day 22) and 336 (Day 29) h post-morning-dosing.

#### Pharmacokinetic assessments

Primary PK parameters assessed for midazolam and its metabolites (1-OH-midazolam, 4-OH-midazolam), caffeine and opelconazole were *C*_max_ and AUC, from time zero to *t*, where *t* is the time of the last quantifiable concentration (AUC_(0−t)_; i.e., AUC_(0−12)_ and AUC_(0−24)_). Secondary PK parameters were AUC_(0−∞)_, *T*_max_, apparent *t*_1/2_ and accumulation ratio (*R_O_*) of opelconazole only.

#### Safety assessments

Safety and tolerability were assessed by monitoring the incidence, nature and severity of adverse events (AEs) as well as by vital-sign measurements, 12-lead ECGs, clinical laboratory testing (haematology, chemistry and urinalysis), physical examination and lung function testing [forced expiratory volume in one second (FEV_1_) and forced vital capacity (FVC)].

#### Statistics

All analyses were conducted in accordance with the study protocol and statistical analysis plan. Data were reported using SAS software (version 9.4 or later). The sample size (*N* = 24) was not selected based on statistical considerations but was considered adequate to reach the objectives of the study and reliably estimate the magnitude and variability of an interaction.

To adjust for any residual caffeine in plasma samples, the estimated contribution of residual, pre-dose caffeine was subtracted from the measured caffeine concentration to produce an ‘adjusted concentration.’ Residual caffeine concentrations were estimated from the baseline concentration and the measured elimination rate in individual participants.

For caffeine, midazolam and the metabolites of midazolam, the geometric mean ratio (GMR) of Day 15 estimates to those obtained on Day 1 was derived by fitting a linear mixed effect model with the log of the parameter as the response, visit as the fixed effect, and participant as the random effect. A DDI would be established if the GMR for AUC_(0–24)_ and for *C*_max_ was increased by a factor of ≥5 (strong inhibitor of CYP3A4 or CYP1A2); increased by ≥2 but <5, (moderate inhibitor), or increased by ≥1.25 but <2, (weak or mild inhibitor).^24^

## Results

### *In vitro* results

#### Assessment of opelconazole as a potential substrate of human CYP isoforms

An *in vitro* reaction phenotyping study assessed which human hepatic CYP isoforms make a significant contribution to the metabolism of opelconazole and found that opelconazole is a substrate for both CYP3A4 and CYP2C9. The relative contributions of CYP3A4 and CYP2C9 were ∼75% and 25%, respectively, when incubated with 0.01 and 0.1 µM opelconazole.

#### Assessment of opelconazole as a potential inhibitor of human CYP isoforms

**Figure 2.**
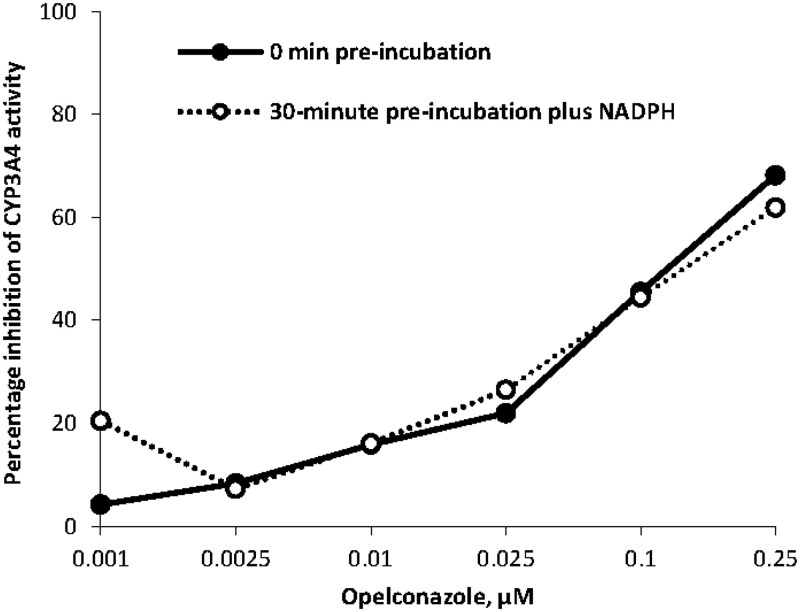
Percentage inhibition of CYP3A4 by opelconazole, using midazolam as a substrate (*in vitro* data). CYP, cytochrome P450.

The only inhibitory interaction observed in the *in vitro* studies with opelconazole was with CYP3A4 substrates. Using a test concentration range of 0.001–0.25 µM (without pre-incubation) opelconazole showed IC_50_ values of 0.123 and >0.25 µM for midazolam and testosterone, respectively. The positive control inhibitor in this study (verapamil) showed satisfactory data. When a 30-min preincubation with opelconazole was included in the test protocol, a time-dependent inhibition of CYP3A4, using midazolam as substrate, was not evident: the IC_50_ was ∼0.15 μM (102 ng/mL; Figure 2). In the testosterone incubations, a small shift in IC_50_ was observed but the inhibition potency (IC_50_ = 0.15 μM for testosterone) in the 30-min preincubations did not have a significant difference with or without NADPH. Overall, it was concluded that opelconazole may act as a reversible inhibitor of CYP3A4 substrates.

#### Assessment of opelconazole as a potential inducer of human CYP1A2, CYP3A4 and CYP2B6

For CYP1A2, opelconazole (≥0.3 μM) caused concentration-dependent increases of 2.72-fold in donor 1 and 1.97-fold in donor 2. For CYP3A4, opelconazole caused an increase in mRNA expression of 2.17-fold but only at 0.3 μM in donor 1; donors 2 and 3 did not reach the 2-fold threshold at any concentration. For CYP2B6, opelconazole did not increase mRNA expression in any of the donors tested. Positive control compounds showed >30-fold greater induction outcomes than with opelconazole (omeprazole for CYP1A2 and rifampicin for CYP3A4). The test results confirm a threshold induction outcome for CYP1A2 and CYP3A4 at an opelconazole concentration of 0.3 μM *in vitro*, but the magnitude of induction was much weaker (at least an order of magnitude less) than those achieved with positive controls.

#### Assessment of opelconazole as a potential substrate or inhibitor of carrier transporters

Opelconazole was not a substrate for any of the transporters tested under the experimental conditions employed. Opelconazole displayed negligible inhibitory interactions with OAT1, OAT3, OCT1, OATP1B1, OATP1B3 and BSEP transporter proteins. At 0.5 μM, opelconazole displayed negligible inhibitory interactions with OCT2 and P-gp; inhibitory interactions from 0.5 μM opelconazole were confirmed with MATE2-K (19%) and BCRP (52%), compared with the corresponding solvent control transporter activity. At 5 μM, inhibitory interactions of opelconazole were confirmed with OCT2 (39%), MATE2-K (52%), P-gp (52%) and BCRP (56%) transporter proteins, relative to solvent controls.

### Phase 1 study of DDIs

#### Pharmacokinetics

**Figure 3.**
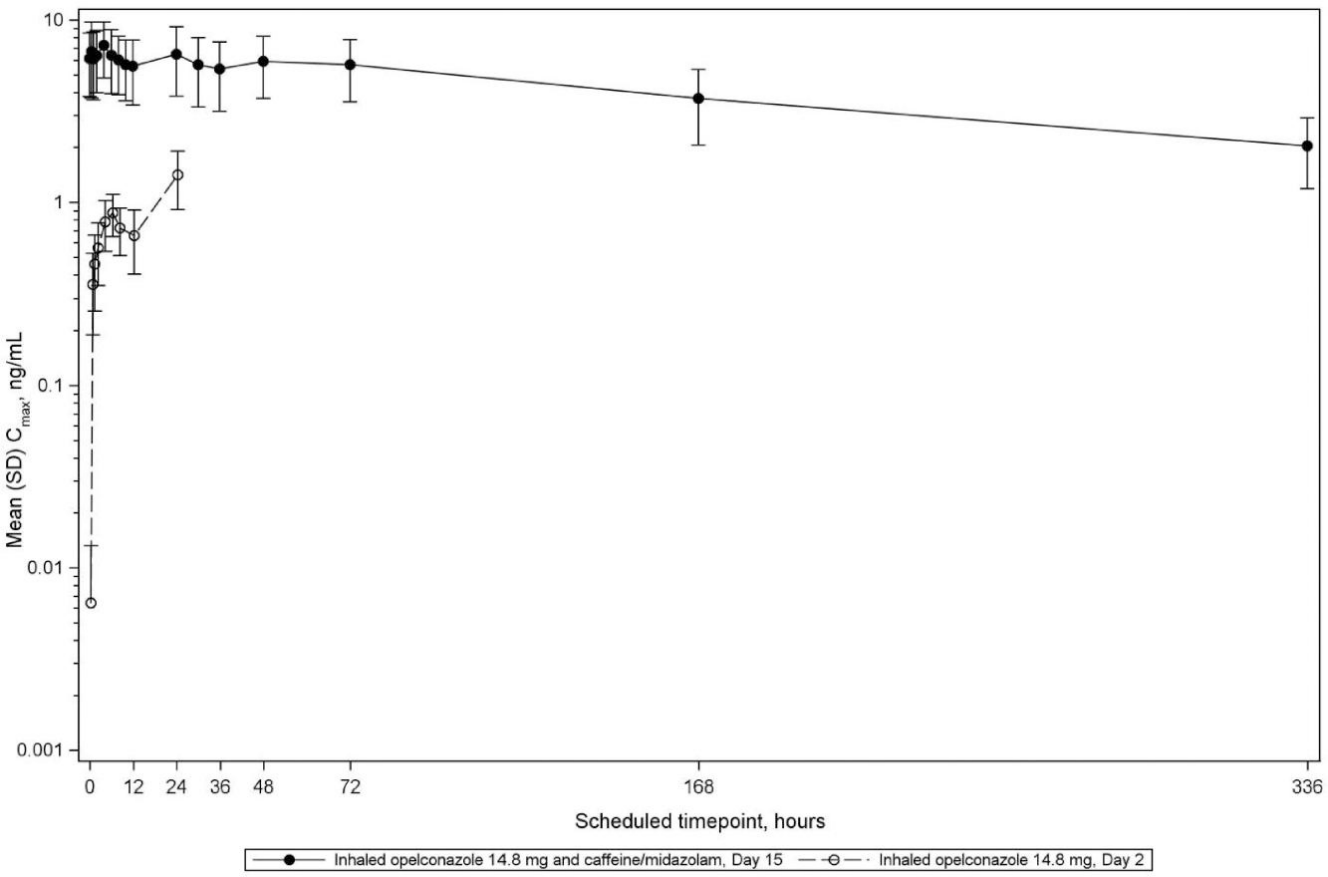
Mean (SD) plasma concentrations of opelconazole (log scale) over time (Phase 1 study). The mean (SD) plasma concentrations of opelconazole are shown after initial dosing on Day 2 (open circles) and from Day 15 after 14 days of twice daily dosing (closed circles). SD, standard deviation.

The PK profile of opelconazole was characterized (Table 2) with results confirming accumulation on repeat dosing with an observed R_O_ (Day 15/Day 2) of 8.9-fold for AUC_0–12h_ and 7.8-fold for *C*_max_. The geometric mean AUC_0–24h_ observed on Day 15 for opelconazole was 136 h·ng/mL (coefficient of variation [CV], 46.7%) and the geometric mean *C*_max_ observed on Day 15 was 7.07 ng/mL (CV, 47.2%; Figure 3).

**Table 2.** Pharmacokinetic parameters of opelconazole

Parameter, unit	Geometric mean (CV%)			
	*n*	Day 2	*n*	Day 15
Opelconazole				
AUC_(0−∞)_, h·ng/mL	—	—	2	1720 (31.2)
AUC_(0–12 h)_, h·ng/mL	24	7.81 (27.8)	24	69.8 (45.4)
AUC_(0–24 h)_, h·ng/mL	24	19.6 (29.4)	24	136 (46.7)
AUC_(0-t)_, h·ng/mL	24	19.9 (29.4)	24	1270 (45.8)
*C*_max_, ng/mL	24	0.91 (29.3)	24	7.07 (47.2)
*T*_max_, median (range), h	24	6.17 (2.17, 12.1)	24	4.17 (0.67, 48.0)
*t*_½_, h	—	—	24	188 (21.4)
*R_O_ C*_max_ Day 15/Day 2	—	—	24	7.77 (46.7)
*R_O_* AUC_(0–12 h)_ Day 15/Day 2	—	—	24	8.93 (38.4)
*R_O_* AUC_(0–24 h)_ Day 15/Day 2	—	—	24	6.97 (35.8)

CV, coefficient of variation; *R_O_*, accumulation ratio.

**Figure 4.**
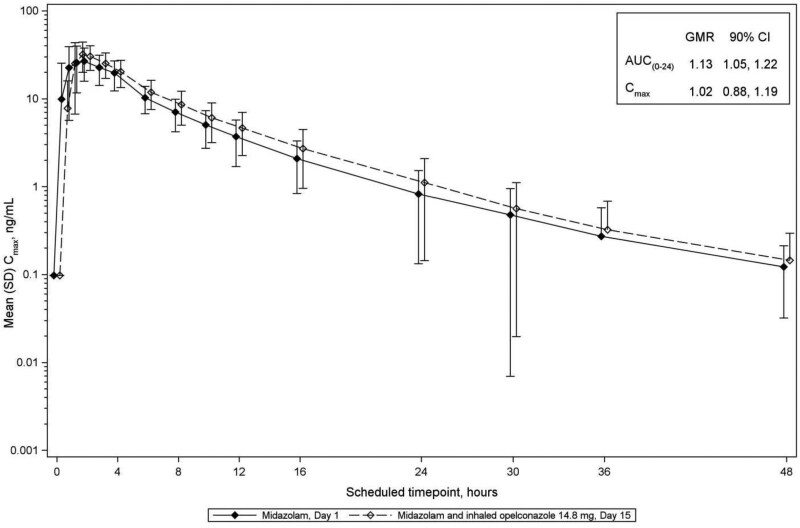
Mean (SD) plasma concentrations for midazolam as dosed on Day 1 and Day 15 (log scale; Phase 1 study). The mean (SD) plasma concentrations of midazolam are shown from Day 1 (closed diamonds) and from Day 15 after 14 days of twice daily dosing of opelconazole (open diamonds). GMR, geometric mean ratio; SD, standard deviation.

**Figure 5.**
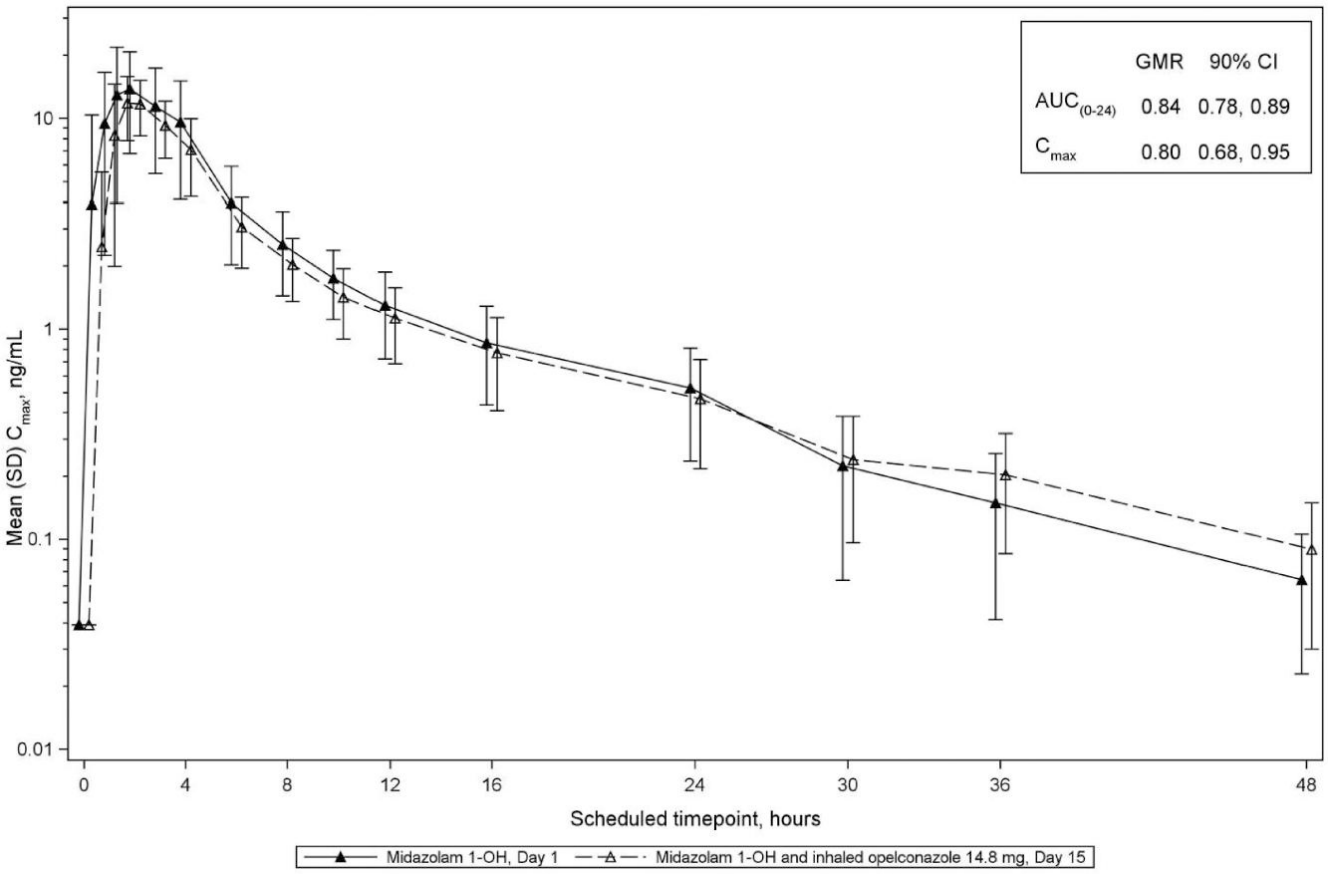
Mean (SD) plasma concentrations for 1-OH-midazolam, a metabolite of midazolam that was dosed on Day 1 and Day 15 (log scale; Phase 1 study). The mean (SD) plasma concentrations of 1-OH-midazolam are shown from Day 1 (closed triangles) and from Day 15 after 14 days of twice daily dosing of opelconazole (open triangles). GMR, geometric mean ratio; SD, standard deviation.

**Figure 6.**
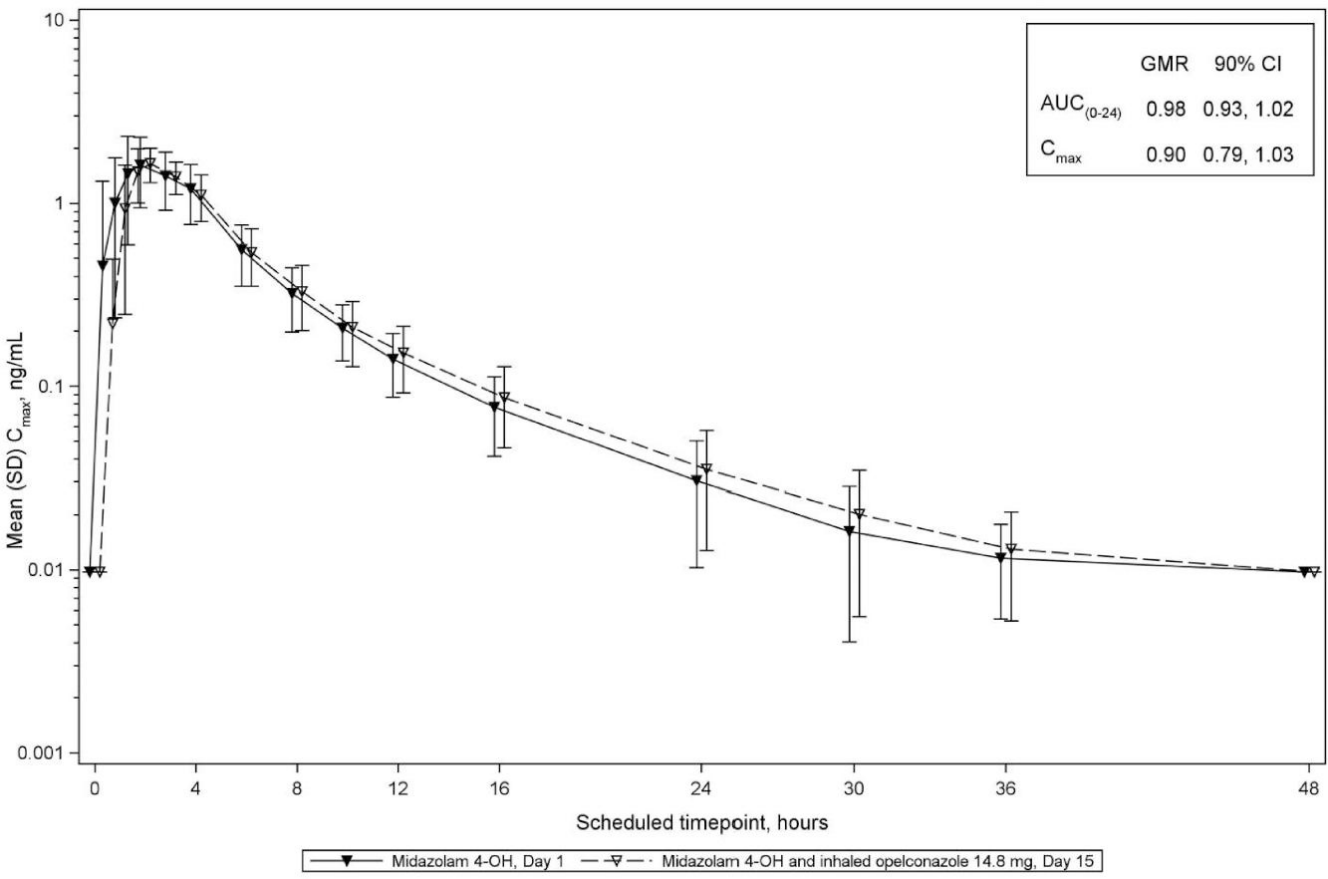
Mean (SD) plasma concentrations for 4-OH-midazolam, a metabolite of midazolam that was dosed on Day 1 and Day 15 (log scale; Phase 1 study). The mean (SD) plasma concentrations of 4-OH-midazolam are shown from Day 1 (closed inverted triangles) and from Day 15 after 14 days of twice daily dosing of opelconazole (open inverted triangles). GMR, geometric mean ratio; SD, standard deviation.

**Figure 7.**
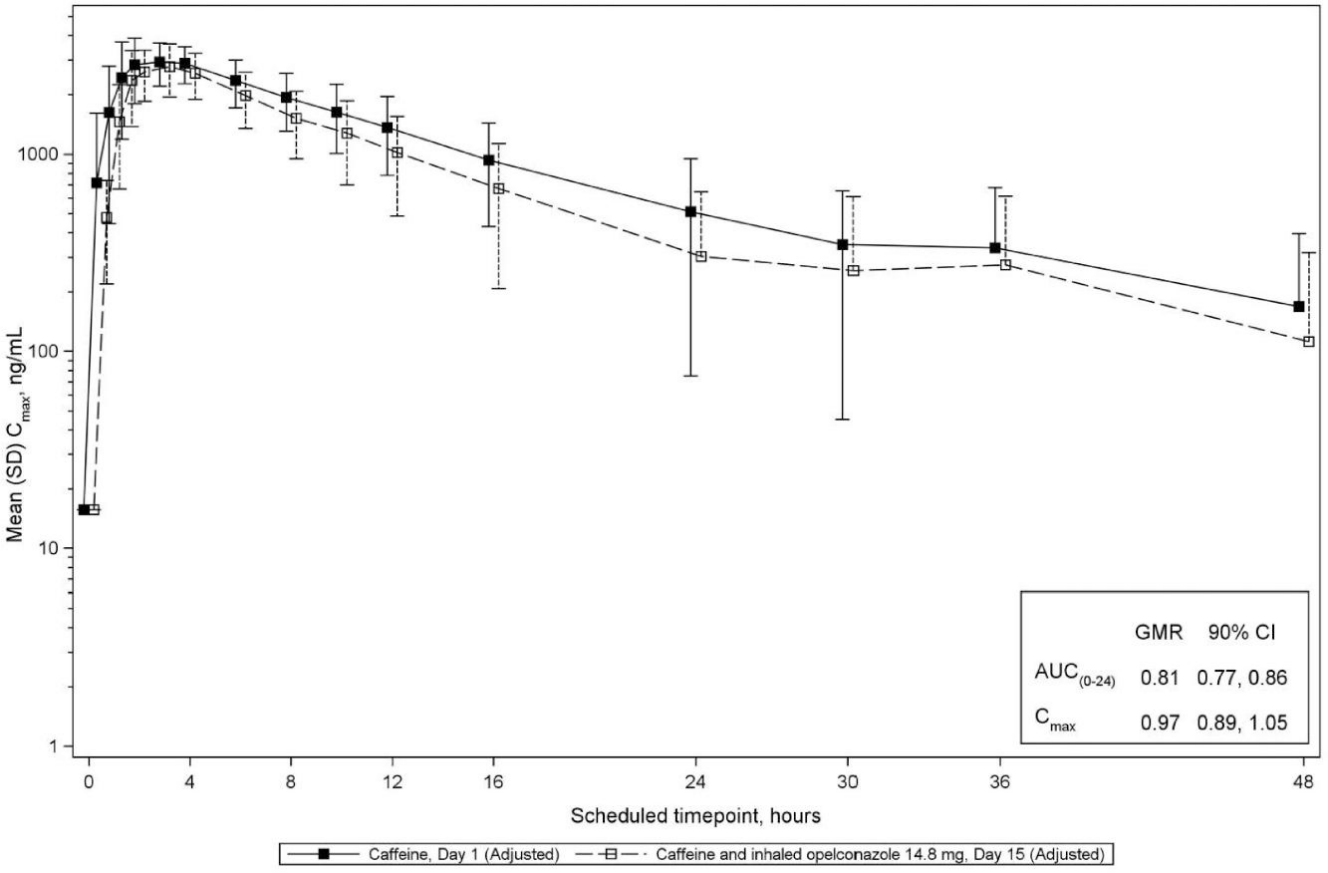
Mean baseline-adjusted caffeine plasma concentrations that was dosed on Day 1 and Day 15 (log scale; Phase 1 study). The mean (SD) plasma concentrations of caffeine are shown from Day 1 (closed squares) and from Day 15 after 14 days of twice daily dosing of opelconazole (open squares). To adjust for any residual caffeine in plasma samples, the estimated contribution of residual, pre-dose caffeine was subtracted from the measured caffeine concentration to produce an ‘adjusted concentration.’ Residual caffeine concentrations were estimated from the baseline concentration and the measured elimination rate in individual participants. GMR, geometric mean ratio; SD, standard deviation.

For midazolam, 1-OH midazolam, 4-OH-midazolam and caffeine, the GMRs and 90% CIs for AUC_0–24 h_ and *C*_max_ were less than the FDA-predefined threshold of 1.25 indicating that opelconazole, at clinically meaningful plasma concentrations which reflect therapeutic concentrations in the lung, had no significant effect on the PK of these probes (Table 3; Figures 4–7). Thus, the potential for inhibition/induction of CYP3A4 or CYP1A2 by opelconazole is considered to be negligible.

**Table 3.** Opelconazole/midazolam/caffeine drug interaction summary

Parameter, unit	Geometric mean*				Geometric Mean ratio, %	90% CI of ratio
	*n*	Day 1	*n*	Day 15		
Midazolam						
AUC_(0–24h)_, h·ng/mL	24	166	24	188	1.13	1.05, 1.22
*C*_max_, ng/mL	24	35.1	24	36.0	1.02	0.88, 1.19
1-OH-Midazolam						
AUC_(0–24h)_, h·ng/mL	24	72.8	24	60.9	0.84	0.78, 0.89
*C*_max_, ng/mL	24	16.7	24	13.4	0.80	0.68, 0.95
4-OH-Midazolam						
AUC_(0–24h)_, h·ng/mL	24	8.92	24	8.70	0.98	0.93, 1.02
*C*_max_, ng/mL	24	1.97	24	1.78	0.90	0.79, 1.03
Caffeine						
AUC_(0–24h)_, h·ng/mL	24	33 200	24	26 900	0.81	0.77, 0.86
*C*_max_, ng/mL	24	3280	24	3170	0.97	0.89, 1.05

#### Safety

No deaths or serious AEs were reported during the study, and none of the AEs were severe. No AEs led to discontinuation of study medications or study withdrawal. No AEs related to laboratory variables were reported. The only AE experienced by more than one participant considered to be related to inhaled opelconazole during exposure to midazolam/caffeine and inhaled opelconazole was headache [experienced by 5/24 participants (20.8%); all recorded as mild]. The other AEs experienced by single participants and considered to be related to opelconazole were abdominal pain, throat irritation and erythema. Two of 5 participants who reported headache required therapeutic intervention (paracetamol). All other AEs resolved spontaneously. No clinically significant changes in lung function were observed at any timepoint during the study. There was no evidence of post-dose acute bronchospasm in any participant who received opelconazole. No AEs of special interest, including bronchospasm, cough, or airway irritation, were reported.

## Discussion

The *in vitro* reaction phenotyping study to assess which human hepatic CYP isoenzymes were involved in the metabolism of opelconazole demonstrated that opelconazole is a substrate for both CYP3A4 and CYP2C9, with relative contributions of about 75% and 25%, respectively. Consequently, it is possible that systemic exposure to opelconazole could be increased by co-administration of a potent CYP3A4 or CYP2C9 inhibitor. However, since opelconazole is a substrate of CYP2C9, this has the potential to mitigate the magnitude of any effect of a co-administered CYP3A4 inhibitor on the systemic PK of opelconazole. As the phenotyping data indicated that CYP3A4 was the principal metabolizing isoform, co-administration of opelconazole with a CYP2C9 inhibitor is unlikely to lead to a significant increase in systemic exposure to opelconazole.

The effect of opelconazole on the activities of CYP isoforms in pooled human liver microsomes was determined *in vitro*. The only inhibitory interaction observed in these studies was with CYP3A4 substrates, with an IC_50_ of ∼0.15 μM (102 ng/mL). Similarly, the potential for opelconazole to act as an inducer of CYP1A2, CYP2B6 and CYP3A4, also determined *in vitro*, confirmed a threshold induction outcome for CYP1A2 and CYP3A4 at an opelconazole concentration of 0.3 μM (204 ng/mL). Subsequent to these *in vitro* DDI assessments, the Phase 1 study in healthy volunteers demonstrated, unequivocally, that at steady state (0.01 μM or 7 ng/mL), opelconazole showed no inhibition or induction of CYP3A4 or CYP1A2, with the GMRs and 90% CIs for AUC and *C*_max_ of the probe agents before and after opelconazole administration being <1.25 (the FDA-predefined threshold). These outcomes are considered to be a direct consequence of the low systemic exposure achieved with multiple inhaled doses of opelconazole.

Carrier transporter protein interactions with opelconazole as a substrate and/or inhibitor were assessed against a full range of uptake and efflux transporters. The findings demonstrated that (1) opelconazole was not a substrate for any transporters tested and (2) moderate to mild inhibition was observed with only four transporters (OCT2, P-gp, MATE2-K and BCRP). As there is negligible oral bioavailability of any swallowed fraction of the opelconazole product evaluated (unpublished data; L.M.R. Cass and L. Berman), it is unlikely that a first-pass inhibition of efflux transporters P-gp and BCRP will occur in the gastrointestinal tract. As the expected clinical steady-state systemic *C*_max_ for opelconazole of ≤0.01 μM (≤6.830 ng/mL) is two to three orders of magnitude lower than the inhibitory concentration of opelconazole on OCT2, MATE2-K, P-gp and BCRP, and, given the previously reported high PPB (independent of concentration) associated with opelconazole, it is considered improbable that interactions with the substrates of these transporters will occur.^17^

A systemic drug interaction, if it were to occur, is not anticipated to alter the antifungal efficacy of inhaled opelconazole since the antifungal effect would be dependent on concentration in the lung resulting from nebulization and lung retention.

Inhaled opelconazole 14.8 mg twice daily was generally well tolerated by participants in the Phase 1 study, and all AEs were mild or moderate in severity. No serious AEs occurred, and no AEs led to study withdrawal by any participants. Respiratory tolerability was good, and no clinically significant changes in lung function (FEV_1_ and FVC) were observed throughout the study, including pre- and post-dosing of inhaled opelconazole.

While triazoles are currently the preferred first-line agents to treat and prevent IPA, systemic triazoles have been associated with class-related DDIs that often limit their use.^9,10,12^ Several CYP enzymes, especially CYP3A4, and the P-gp membrane transporter metabolize and are inhibited by triazoles.^10^ The *in vitro* and Phase 1 data reported here suggest that inhaled opelconazole has the potential to overcome some of the limitations associated with systemic triazoles, especially with respect to DDIs. These findings may be of particular clinical relevance for patients receiving chemotherapeutic or immunosuppressant therapies, many of which are metabolized by CYP3A4.^14^

Opelconazole, formulated for inhalation delivery, is characterized by high lung concentrations and lung retention, low systemic exposure at steady state (*C*_max_: 0.01 μM or 7 ng/mL) and high PPB.^17,21^ Inhaled opelconazole was generally well tolerated in clinical trials, with a negligible risk of DDIs. As opelconazole is a substrate for CYP3A4, recently completed physiologically-based PK modelling indicated a weak interaction (≥1.25 to <2-fold increase) if opelconazole were to be co-administered with a strong 3A4 inhibitor such as itraconazole (unpublished data; L.M.R. Cass and L. Berman).^24^ The resulting potential increase in systemic concentrations is expected to be in the single-digit ng/mL range and with 97% PPB, the clinical relevance in terms of safety and tolerability is expected to be negligible.^17^ These characteristics indicate that therapeutic drug monitoring (TDM) should not be necessary for inhaled opelconazole. In contrast, TDM is currently recommended for patients receiving systemic triazoles or therapies that interact with triazoles once steady state has been reached.^9,10^

In conclusion, the potential for interaction of opelconazole with CYP3A4 or CYP1A2 substrates during concomitant use is negligible, and opelconazole use is unlikely to be associated with CYP-mediated DDIs in the clinic, especially CYP3A4-mediated DDIs. The ongoing inhaled opelconazole clinical programme will further elucidate its therapeutic potential to treat IPA in clinical practice.

## Transparency declarations

J.A. is a consultant to and has share options in Pulmocide Ltd. L.B. is an employee of and has share options in Pulmocide Ltd. R.J.M.B. serves as a consultant to Basilea Pharmaceutica Ltd, has served as consultant to Astellas Pharma, Inc., F2G, Amplyx, Gilead Sciences, Merck Sharp & Dohme Corp., Mundipharma, and Pfizer, Inc. and has received unrestricted research grants from Astellas Pharma, Inc., Gilead Sciences, Merck Sharp & Dohme Corp. and Pfizer, Inc. All contracts and payments were through his institution. L.M.R.C. is an employee of and has share options in Pulmocide Ltd. L.G. has served as a consultant to Pulmocide Ltd. M.H. has served as a consultant to Pulmocide Ltd. U.M. was an employee of and has share options in Pulmocide Ltd. J.M. has served as a consultant to Pulmocide Ltd. J.A., L.B., L.M.R.C. and U.M. developed the research ideas, study designs, methodologies and protocols. J.A., L.M.R.C., L.G., M.H. and J.M. contributed to data collection. L.G. and J.M. provided statistical analysis of the data. All authors reviewed and analysed the data sets from the studies. J.A., L.B. and L.M.R.C. contributed to the development of the original draft of the manuscript. All authors reviewed and revised the original and subsequent drafts of the manuscript. Medical writing support was provided by Kathy Boltz, PhD, and Emily Donovan and was supported by Pulmocide Ltd.
